# The psychiatric risk gene *BRD1* modulates mitochondrial bioenergetics by transcriptional regulation

**DOI:** 10.1038/s41398-022-02053-2

**Published:** 2022-08-08

**Authors:** Veerle Paternoster, Cagla Cömert, Louise Sand Kirk, Sanne Hage la Cour, Tue Fryland, Paula Fernandez-Guerra, Magnus Stougaard, Jens Randel Nyengaard, Per Qvist, Peter Bross, Anders Dupont Børglum, Jane Hvarregaard Christensen

**Affiliations:** 1 The Lundbeck Foundation Initiative for Integrative Psychiatric Research, iPSYCH, Aarhus, Denmark; 2 Centre for Integrative Sequencing, iSEQ, Aarhus University, Aarhus, Denmark; 3 Department of Biomedicine, Aarhus University, Aarhus, Denmark; 4 Center for Genomics and Personalized Medicine, CGPM, Aarhus University, Aarhus, Denmark; 5 Research Unit for Molecular Medicine, Department of Clinical Medicine, Aarhus University and Aarhus University Hospital, Aarhus, Denmark; 6 Department of Pathology, Aarhus University Hospital, Aarhus, Denmark; 7 Department of Clinical Medicine, Aarhus University, Aarhus, Denmark; 8 Core Center for Molecular Morphology, Section for Stereology and Microscopy, Department of Clinical Medicine, Aarhus University, Aarhus, Denmark

**Keywords:** Schizophrenia, Pathogenesis

## Abstract

Bromodomain containing 1 (*BRD1)* encodes an epigenetic regulator that controls the expression of genetic networks linked to mental illness. BRD1 is essential for normal brain development and its role in psychopathology has been demonstrated in genetic and preclinical studies. However, the neurobiology that bridges its molecular and neuropathological effects remains poorly explored. Here, using publicly available datasets, we find that BRD1 targets nuclear genes encoding mitochondrial proteins in cell lines and that modulation of *BRD1* expression, irrespective of whether it is downregulation or upregulation of one or the other existing BRD1 isoforms (BRD1-L and BRD1-S), leads to distinct shifts in the expression profile of these genes. We further show that the expression of nuclear genes encoding mitochondrial proteins is negatively correlated with the expression of *BRD1* mRNA during human brain development. In accordance, we identify the key gate-keeper of mitochondrial metabolism, Peroxisome proliferator-activated receptor (PPAR) among BRD1’s co-transcription factors and provide evidence that BRD1 acts as a co-repressor of PPAR-mediated transcription. Lastly, when using quantitative PCR, mitochondria-targeted fluorescent probes, and the Seahorse XFe96 Analyzer, we demonstrate that modulation of *BRD1* expression in cell lines alters mitochondrial physiology (mtDNA content and mitochondrial mass), metabolism (reducing power), and bioenergetics (among others, basal, maximal, and spare respiration) in an expression level- and isoform-dependent manner. Collectively, our data suggest that BRD1 is a transcriptional regulator of nuclear-encoded mitochondrial proteins and that disruption of BRD1’s genomic actions alters mitochondrial functions. This may be the mechanism underlying the cellular and atrophic changes of neurons previously associated with BRD1 deficiency and suggests that mitochondrial dysfunction may be a possible link between genetic variation in *BRD1* and psychopathology in humans.

## Introduction

Psychiatric disorders comprise a heterogeneous group of conditions collectively characterized by changes in patterns of thoughts, emotions, and behaviors. Their clinical profile is shaped by a range of environmental exposures [1] and molecular genetic studies have identified common and rare genetic variants that contribute to mental illness [2]. Overlap in epidemiological risk factors and shared genetic burden further suggests that psychiatric disorders share a common etiology [3]. Although recent research has outlined numerous putative biological underpinnings in psychiatric disorders [4], the majority of the psychiatric risk loci remain to be translated into pathobiology.

The Bromodomain containing 1 gene, *BRD1*, has been associated with mental illness in several genetic studies of common variants [5–12], including genome-wide association to schizophrenia (SZ) [10]. *BRD1* risk alleles are associated with increased *BRD1* promoter DNA methylation [13] and with reduced expression of *BRD1* in the blood in the general population [14]. In addition, rare disruptive variants in *BRD1* have been reported in both SZ and autism spectrum disorder (ASD) cases [9, 15–17]. Patients with 22q13 microdeletions, which among other genes span *BRD1*, further present with varying constellations of neurological, somatic, and behavioral symptoms including among many others neonatal hypotonia, developmental delay, intellectual disability, ASD, speech delay, elevated pain threshold, and seizures [18, 19]. Intriguingly, these patients can also present with psychiatric disorders like OCD, affective disorder, and atypical bipolar [20], whereas SZ is not as common [21, 22].

*BRD1* encodes a scaffold protein that interacts with epigenetic modifiers affecting histone acetylation and histone H3 N-tail clipping and hereby modulates the transcription of a comprehensive set of genes implicated in brain development and mental disorder risk [14, 23–25]. Importantly, BRD1 has different isoforms (BRD1-L and BRD1-S) that appear to govern overlapping but distinct chromatin interactomes [23]. In line with an important role in spatio-temporal gene regulation in the central nervous system, the expression of *BRD1* and its splice variants is highly regulated during brain development [6], stem cell differentiation [26], and upon external stimuli like chronic restraint stress [27] and electroconvulsive seizures in rats [28] as well as upon administration of commonly used mood stabilizers in cell lines [13]. Emphasizing the importance of BRD1-regulated transcription in normal brain development and function, *BRD1* has, in the general population, been associated with the surface area of the cerebral cortex as measured by magnetic resonance imaging [29, 30], and haploinsufficient *Brd1*^*+/−*^ mice are characterized by changes in neurochemistry, brain-, and synapse morphometry accompanied by behavioral alterations and cognitive impairments with broad translational relevance to psychiatric disorders [14, 31–33]. Interestingly, brain transcriptomic and proteomic profiling of *Brd1*^+/−^ mice has previously hinted at mitochondrial dysfunction in several brain tissues [14, 32, 34], suggesting that BRD1, and potentially its isoforms, might be important in epigenetic regulation of mitochondrial function, cellular metabolism, and bioenergetics.

In the present study, we assess the role of BRD1 in mitochondrial biology. Through integrative bioinformatics analyses of human datasets and in vitro studies in cell lines, we provide evidence linking hampered BRD1-regulated isoform-specific gene expression to mitochondrial dysfunction.

## Materials and methods

### Publicly available datasets

The following publicly available datasets were used throughout the manuscript: nuclear-encoded mitochondrial (nMT) proteins from MitoCarta 3.0 [35]. Developmental brain transcriptome from Brainspan (www.brainspan.org); BRD1-S and BRD1-L genomic targets in HEK293T cells [23]. Expression microarray analyses of HEK293T cells with siRNA induced BRD1 knockdown (BRD1-KD) or overexpression of the BRD1-S or BRD1-L isoforms [23]. Gene lists are given in Supplementary Table S1.

### Enrichment analysis

We downloaded the developmental brain transcriptome from Brainspan (www.brainspan.org) for gene co-expression and spatiotemporal analysis. Only protein-coding genes with a unique gene symbol were retained. Genes with missing data and genes with an overall coefficient of variance <0.1 were filtered to avoid genes with little or no information concerning the spatiotemporal dynamics, resulting in the inclusion of expression information for 18,828 transcribed genes. We then divided the expression into 32 spatiotemporal intervals consisting of eight temporal intervals (p1–p8) and four brain regions (r1–r4) as described previously [23, 36]. Spearman correlation coefficients were calculated as estimates for co-expression for the whole dataset as well as for the 32 intervals. The R script is freely available on Github (https://github.com/veerlepaternoster/BrainspanCorrelation). A total of 961 nMT transcripts (5.1%), were present in the curated Brainspan expression dataset. The similarity in the distribution of correlation values was assessed by the Mann–Whitney *U* test. Enrichment of nMT genes in respectively, highly positively correlated (*r* > 0.5), highly negatively correlated (*r* < −0.5) or BRD1 genomic target subsets was tested by Chi-square test or Fisher’s exact test, depending on group size. Since each of the 32 spatiotemporal intervals contained both independent data points (values from individual donors) and dependent data points (values from different regions from the same donor), we applied a conservative correction for multiple testing (the Bonferroni method) when appropriate to reduce the number of false positives. GO term enrichment calculation was done using the DAVID Bioinformatics Resources 6.8 [37] using the highly negatively correlated subset as gene lists and the curated Brainspan gene list as the background list. Enrichment of transcription factor binding was assessed using the Enrichr analysis tool (ENCODE and ChEA Consensus TFs from ChIP-X) [38, 39] with standard settings.

### Cell lines and culture

The CRISPR/Cas9 system was used to establish a heterozygous *BRD1* knock out (BRD1^CRISPRex6*/+*^) cell line (Supplementary Information). In addition, three previously generated HEK293T cell lines were used: HEK293T (control) and stably overexpressing His6- and V5-tagged BRD1 isoforms (BRD1-L or BRD1-S) [23, 40]. Cell lines were grown in DMEM medium (Sigma, Saint Louis, MO, USA) supplemented with 10% fetal calf serum (Sigma), 50 mg/L L-glutamine (Gibco, Paisley, UK), 50 mg/L penicillin (Gibco), 50 mg/L streptomycin (Gibco) at standard culture conditions, 37 °C in 5% CO_2_.

### Nuclear receptor 10-pathway reporter array

The transcriptional drive of BRD1^CRISPRex6/+^ cells was investigated in a Cignal Finder Nuclear Receptor 10-Pathway Reporter Array (Qiagen, Hilden, Germany). Briefly, four different colonies of BRD1^CRISPRex6/+^ and four colonies of naïve HEK293T cells were co-transfected with plasmids carrying a firefly luciferase reporter coupled to individual nuclear receptor transcriptional response elements (12.5 ng/1000 cells) and a plasmid carrying a renilla reporter coupled to a constitutively active cytomegalovirus (CMV) promoter (12.5 ng/1000 cells) using 0.2 μL TurboFect^TM^ Transfection Reagent (Thermo Scientific, Waltham, MA, USA). Cells were subsequently cultured for 48 h followed by cell lysis. Firefly and renilla luciferase activity was measured using the Dual-Luciferase® Reporter (DLR^TM^) Assay System (Promega, WI, USA) on a MicroLumat Plus LB96V (Berthold Technologies, Bad Wildbad, Germany) according to the manufacturer’s protocol. This was repeated in an independent, identical setup.

### Quantitative real-time PCR

DNA and RNA were extracted from frozen cell pellets using the AllPrep DNA/RNA extraction kit (Qiagen) according to the manufacturer’s recommendations. Concentrations were measured using the Nanodrop 1000 version 3.7.1 (Thermo Scientific). The quality of DNA and RNA was assessed by the A260/280 O.D. ratio. For DNA, we accepted an A260/280 O.D. ratio of ~1.8, for RNA a ratio of ~2.0. The quality of RNA was further assessed by inspection of the integrity and relative abundancies of the 28S and 18S ribosomal RNA using gel electrophoresis. cDNA was synthesized from 1 µg total RNA using the iScript™ cDNA Synthesis Kit (Biorad, Hercules, CA, USA) according to manufacturer’s recommendation.

PCR amplification and simultaneous detection of DNA or cDNA template was performed using the LightCycler480 SYBR Green Master (Roche, Basel, Switzerland). The quality of the primers was assessed by primer efficiency (1.8 < *E* < 2.1) according to MIQE guidelines [41]. The PCR reaction was performed using 40 cycles of denaturation (95 °C), annealing (60 °C), and extension (72 °C). After the amplification steps, a melting step was included to detect possible unspecific amplification products. For quantification of gene expression based on cDNA, samples were diluted 1:40 and raw relative abundances compared to a standard curve (four serial dilutions of a pool of all cDNA samples (1:5, 1:25, 1:125 and 1:625)) were extracted by the LightCycler480 software (Roche) before statistical analysis. We included five reference genes (*GAPDH, POLR2, HPRT, RPS,* and *PGK1*) in the analysis and selected for normalization the most stable combination of two of these by the Normfinder Software [42].

For quantification of mitochondrial DNA content, samples were diluted to 25 ng/μL. Two reference amplicons located in the nuclear genome were included (*GAPDH* and *SLC34A2*) and the mean signal was used as reference. Relative mitochondrial DNA content to each of the reference amplicons was calculated separately as follows:

$${\rm{Relative}\;\rm{mitochondrial}\;\rm{DNA}\;\rm{content}} = 2 \ast 2^{(\rm{nuclear}\;C_t - {\rm{mitochondrial}}\;C_t)}.$$

Primer sequences are given in Supplementary Table S2.

### Image cytometry for cellular and mitochondrial phenotyping

Image cytometry with an NC-3000 Image Cytometer (ChemoMetec, Allerod, Denmark) was used for cellular and mitochondrial phenotyping. A minimum number of 5000 events were analyzed in each experiment. The following assays were executed according to the manufacturer’s recommendations: cell count (Acridine orange and DAPI (ChemoMetec)), mitochondrial membrane potential (JC-1 and DAPI (ChemoMetec)), thiol redox status (VitaBright-48, Propidium iodide, and acridine orange (ChemoMetec)). Mitochondrial superoxide levels (MitoSOX™ (Thermo Scientific) and Hoechst-33342 (ChemoMetec)) were measured as described earlier [43]. To measure mitochondrial mass, 100 nM fluorescent dye MitoTracker™ Green FM (Invitrogen, Carlsbad, CA, USA) diluted in Hank’s balanced salt solution (HBSS) (Invitrogen) was added to cells for incubation for 30 min at 37 °C. After removing excess dye, the cells were collected and a 1:1000 dilution of RedDot2 (Biotium, Fremont, CA, USA) (concentration not specified) was added to each sample before measurement. The NucleoView NC-3000 software (ChemoMetec) was used for data analysis.

### Calcium buffering capacity

Mitochondria were extracted from 20 × 10^6^ cells using the Mitochondria Isolation Kit for Cultured Cells (Thermo Scientific) and redissolved in 50 µL respiration buffer (RB) (2 × RB: 274 mM KCl, 20 mM HEPES-KOH pH 7.4, 5 mM MgCl_2_, 6 mM KH_2_PO_4_-KOH pH 7.4, 50 µM EDTA) with 5 mM succinate, 5 mM glutamate, 5 mM malate, and calcium green-5 N (Thermo Scientific)) [44]. Mitochondrial content was estimated based on protein content and quantified based in the absorbance at 280 nm using the Nanodrop 1000 version 3.7.1 (Thermo Scientific). Calcium buffering capacity of 90 µg mitochondria was determined as published elsewhere by measuring changes in fluorescence after repeatedly adding 40 µM Ca^2+^ [44]. The experiment was repeated twice.

### Mitochondrial bioenergetics measurements

17,000 cells were seeded in each well of a Cell-tak (Corning Life Sciences, MA, USA) coated 96-well XF96 cell culture microplate (Seahorse Bioscience, Agilent Technologies, Santa Clara, CA, USA) and incubated in culture media for 16 h at 37 °C in a humidified atmosphere of 5% CO_2_. One hour before the assay, culture medium was changed into XF base medium (Seahorse Bioscience) supplemented with 2 mM glutamine, 1 mM sodium pyruvate, and 10 mM glucose and the culture plate was incubated in a non-CO_2_ incubator at 37 °C. To assess mitochondrial respiration in cells, the XF Cell Mito Stress Test (Seahorse Bioscience) was used with an XFe96 extracellular flux analyzer (Seahorse Bioscience). Oxygen consumption rate (OCR) and Extracellular Acidification Rate (ECAR) were measured three sequential times simultaneously in each well, at baseline as well as after the successive injection of 1 µM Oligomycin (inhibitor of complex V) or 350 nM FCCP (uncoupler), and 0.5 µM rotenone (inhibitor of complex I)/antimycin A (inhibitor of complex III) (Supplementary Fig. S1). Data was analyzed using the Wave software version 2.6.0 (Seahorse Bioscience). The experiment was repeated three times on independently cultured cells. To calculate bioenergetics parameters (Supplementary Table S3), the following time points per interval were chosen according to the manufacturer’s recommendations: time point T_3_ for interval 1 (baseline), time point T_4_ for interval 2 (FCCP or Oligomycin), time point T_9_ for interval 3 (rotenone/antimycin A). Cell lines were compared using a linear model in R (*lmer* function in the ‘lme4’ package [log10(OCR) ~ is.BRD1.KO + is.BRD1.L + is.BRD1.S + (1|plateID)]) including the repeated experiments as random variable. Significant differences between genotypes were calculated using the ‘lmerTest’ package in R.

## Results

### BRD1 targets and regulates nuclear genes encoding mitochondrial proteins

In order to assess if BRD1 is a putative transcriptional regulator of nuclear-encoded mitochondrial (nMT) proteins [45–47], we exploited previously published datasets on BRD1’s chromatin interactome and global gene expression from HEK293T cells either stably overexpressing BRD1 isoforms (BRD1-L or BRD1-S) or transiently underexpressing *BRD1* (BRD1-KD) [23, 40]. BRD1-KD cells are underexpressing both the BRD1-L and BRD1-S isoform. Among 19,234 protein-coding genes in the human nuclear genome, we identified 1123 (5.8%) as nMT genes (as defined by MitoCarta 3.0 [35]). When only looking at protein-coding genes within the chromatin interactome of BRD1-S (*n* = 976) and BRD1-L (*n* = 507), respectively 8.1% and 9.9% of those were nMT genes, representing a significant enrichment (Chi-square test, *p*_BRD1-S_ = 0.002 and *p*_BRD1-L_ = 0.0001).

**Fig. 1:**
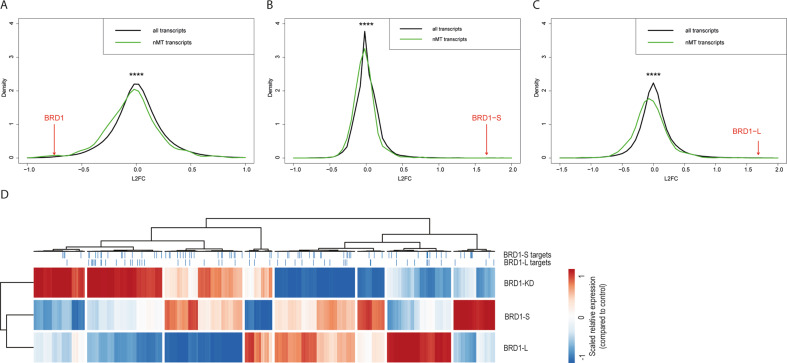
Expression of nuclear-encoded mitochondrial (nMT) transcripts upon *BRD1* under- or overexpression. **A**–**C** The expression of nMT transcripts in HEK293T cells with **A** *BRD1* underexpression (BRD1-KD) or **B** overexpression of BRD1-S or **C** overexpression of BRD1-L isoforms were compared to the parental HEK293T control cell line by plotting log2 fold change (L2FC) of all transcripts (black, *n*_all_ = 17,328) and nMT transcripts (green, *n*_nMT_ = 954). In all three comparisons, a significant shift in the expression profiles towards negative L2FC for nMT transcripts was observed, as determined by the Mann–Whitney *U* test. ****p **<** 0.0001 (*p* **<** 10^−8^). Red arrows indicate expression levels of respectively, total *BRD1* (BRD1), *BRD1-S,* and *BRD1-L* splice variants. **D** Relative changes in mRNA levels for nMT genes in BRD1-KD, BRD1-S, and BRD1-L cells compared to naïve HEK293T control cells. Columns represent nMT genes and rows represent BRD1-KD, BRD1-S, and BRD1-L cells. L2FC values are scaled (unit variance scaling) and column-centered. Clustering is based on correlation. Top panel marks, respectively, BRD1-S and BRD1-L target genes.

We then compared the overall changes in the expression profile of nMT transcripts to the profiles of all transcripts in BRD1-L, BRD1-S, and BRD1-KD cells and found a significant shift in the profile towards a negative fold change (downregulation) for nMT transcripts (BRD1-KD: *p* = 2 × 10^−9^; BRD1-L: *p* = 7 × 10^−17^; and BRD1-S: *p* = 3 × 10^−14^, Fig. 1A–C). Thus, irrespective of whether *BRD1* is underexpressed in cells or one or the other BRD1 isoform is upregulated, similar overall shifts in expression profiles are observed.

Interestingly, when inspecting the different expression profiles of nMT genes in BRD1-L, BRD1-S, and BRD1-KD cells by cluster analyses based in log2 fold changes (compared to naïve HEK293T control cells) (Fig. 1D), we observed distinct patterns of expression changes in each of the three types of cells, though with BRD1-L and BRD1-S clustering together (and apart from the BRD1-KD cells). Particularly, it seems that for the majority of nMTs (52%), the direction of fold changes were opposite in BRD1-S and BRD1-L cells compared to BRD1-KD cells, and overall, fold changes were never in the same direction for all three types of cells. We further observe that genes in the BRD1-S and BRD1-L chromatin interactomes (their genomic targets) are present in all the different gene clusters (Fig. 1D, upper panel), and that no obvious correlation exists between the density of targets and expression fold changes in the *BRD1* under- or overexpressing cells compared to control cells.

### The expression of nuclear genes encoding mitochondrial proteins is negatively correlated with *BRD1* expression in the developing human brain

**Fig. 2:**
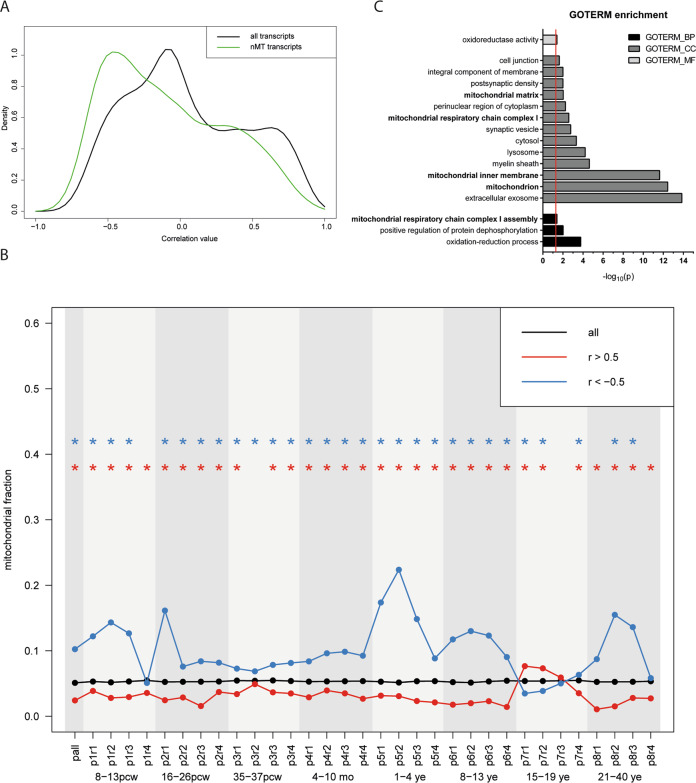
Spatiotemporal analysis of the correlation between nMT transcripts and *BRD1* expression. **A** Expression of nMT transcripts (green) is negatively correlated with *BRD1* expression in the developing human brain compared to the overall gene expression (black). *P*-value as determined by Mann–Whitney *U* test. **B** The fraction of nMT transcripts among all transcripts (black) and among positively (red) and negatively (blue) correlated transcripts is shown for all time points and regions combined (pall) and for each interval (p1r1-p8r4). *P*-value as determined using Chi-square test or Fisher’s Exact test (depending on sample size), where the blue and red asterisks (*) denote *p* < 0.05 after correction for multiple testing by the Bonferroni method. Temporal intervals (P) were grouped as follows: P1 included pcw 8–13 (first trimester), P2 included 16–26 pcw (second trimester), P3 included 35–37 pcw (third trimester), P4 included 4–10 months, P5 included 1–4 years, P6 included 8–13 years, P7 included 15–19 years, and P8 included 21–40 years. Brain regions (R) were grouped as follows: R1 included the posterior inferior parietal cortex, primary auditory cortex, primary visual cortex, superior temporal cortex, and inferior temporal cortex; R2 included the primary somatosensory cortex, primary motor cortex, orbital prefrontal cortex, dorsolateral prefrontal cortex, medial prefrontal cortex, and ventrolateral prefrontal cortex; R3 included the striatum, hippocampus, and amygdala; and R4 included the mediodorsal nucleus of the thalamus, and cerebella cortex. Abbreviations: post-conception week (pcw); months (mo); years (ye). **C** GOTERM enrichment analyses. Negatively correlated transcripts were enriched for mitochondria-related GOTERMs (highlighted in bold). *P*-value is presented as EASE Score, a modified Fisher Exact *P*-value and corrected for multiple testing by the Bonferroni method. Red line at *p*_adjusted_ = 0.05.

While molecular studies in preclinical models have previously indicated a link between expression levels of *BRD1* and mitochondrial function in brain tissue [14, 32, 34], the role of BRD1 as a regulator of mitochondrial gene networks in the developing human brain has not been assessed. Hence, we explored whether the expression of *BRD1* is correlated with the expression of nMT transcripts in the developing human brain. Particularly, we compared expression correlation across 13 developmental stages and 26 different brain regions of *BRD1* and all protein-coding transcripts as well as *BRD1* and nMT transcripts in the Brainspan dataset (*n*_total_ = 18,828). We found that the correlation values of all protein-coding transcripts center on zero (mode = −0.07), indicating no general correlation in expression of protein-coding genes with *BRD1* expression. However, when we compared the distribution of correlation values for nMT transcripts (*n* = 961), we observed a significant shift towards negative correlation (mode = −0.57, *p* = 9 × 10^−31^) (Fig. 2A).

Next, we considered if this negative correlation was present at all developmental stages (p1–p8) of all brain regions (r1–r4) (Fig. 2B) and we calculated the fractions of nMT transcripts in all spatiotemporal intervals. Between 5.1 and 5.5% of all transcripts were nMT transcripts in each interval. When looking only among the negatively correlated transcripts (*r* < −0.5), we found a significant enrichment of nMT transcripts in 28 out of 33 spatiotemporal intervals (fraction of nMT transcripts between 6.3 and 22%) and a significant underrepresentation in 2 out of 33 intervals (fraction of nMT transcripts between 3.4 and 3.8%). Furthermore, nMT transcripts were significantly underrepresented among positively correlated transcripts (*r* > 0.5) in 28 out of 33 spatiotemporal intervals (fraction of nMT transcripts between 1.1 and 3.9%), and significantly overrepresented in 2 out of 33 spatiotemporal intervals (fraction of nMT transcripts 7.3 and 7.6%). Supporting a role for BRD1 as a transcriptional regulator of nMT genes, GOTERM enrichment analysis of all genes with *r* < −0.5 (negatively correlated transcripts in the overall analysis of all brain regions and time points) as input gene set (*n* = 1757) found a significant enrichment of genes annotated to mitochondrial processes (Fig. 2C), including both general and highly specific terms. Particularly, the enrichment of two terms GO:0032981 (mitochondrial respiratory chain complex I assembly) and GO:0005747 (mitochondrial respiratory chain complex I) could indicate a link between complex I of the electron transport chain and BRD1.

### Identification of potential BRD1 co-factors

Next, we wanted to identify potential co-factors participating in the BRD1-mediated regulation of nMT transcripts. We repeated the correlation analyses of human brain expression datasets now analyzing the four epigenetic modifying enzymes that have been identified as protein-protein interaction partners of BRD1: KAT5, KAT7, KMT5B, and DNMT1 [23, 24]. When we compared the expression of nMT transcripts with the expression of these four enzymes respectively, we observed a pronounced shift towards negative correlations with KMT5B (Δmode_nMT-all_ = −0.65, *p* = 1 × 10^−20^) and DNMT1 (Δmode_nMT-all_ = −0.46, *p* = 6 × 10^−29^), while this shift was much smaller with KAT7 (Δmode_nMT-all_ = −0.13, *p* = 8 × 10^−9^) or not significant with KAT5 (Δmode_nMT-all_ = 0.05, *p* = 0.078) (Supplementary Fig. S2). The expression correlation profile for KMT5B and DNMT1 shows high similarity with the expression correlation profile found for *BRD1*, suggesting that the expression of a subset of nMT transcripts may be regulated by the catalytic activity of KMT5B and/or DNMT1 in complex with BRD1.

**Fig. 3:**
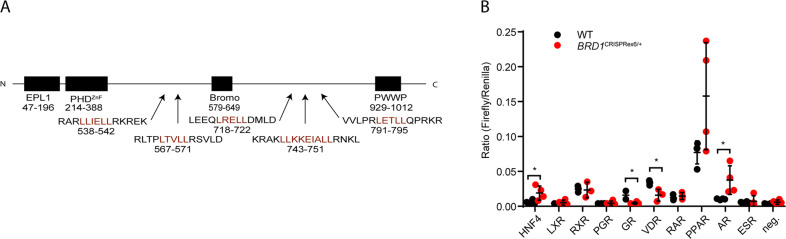
Analysis of the transcriptional drive of hormone response elements (HRE) in BRD1^CRISPRex6/+^ cells. **A** Domains and nuclear receptor binding sites in BRD1. Top: Enhancer of polycomb-like, N-terminal domain (EPL1); Plant homeodomain finger (PHD^ZnF^); Bromodomain (Bromo); Pro-Trp-Trp-Pro (PWWP). Bottom: Amino acid sequences (one-letter code) containing putative nuclear receptor (NR) binding sites (4 co-activators (LXXLL) and 1 co-repressor (LXXIXXL)). Pink letters indicate the putative NR binding sites. Amino acid numbering according to BRD1-S. **B** Transcription from promoters containing HREs recognized by respectively: HNF4 (hepatocyte nuclear factor); LXR (liver receptor); RXR (retinoid X receptor); PGR (progesterone receptor); GR (glucocorticoid receptor); VDR (vitamin D receptor); RAR (retinoic acid receptor); PPAR (peroxisome proliferator-activated receptor); AR (androgen receptor); and ESR (estrogen receptor) as well as a TATA box promoter (negative control (neg)) was tested in four distinct BRD1^CRISPRex6/+^ and four WT HEK cell lines by a dual luciferase-based array. *P*-value as determined by Student’s *t*-tests. *p* < 0.05 (*). Transcription from the PPAR HRE (PPRE)-containing promoter was near significantly increased (*p* = 0.085) in BRD1^CRISPRex6/+^ cells.

To identify potential co-transcription factors of BRD1, participating in the regulation of nMT gene expression, we performed an enrichment analysis to identify transcription factors that bind upstream of the subset of nMT genes that are negatively correlated to *BRD1* expression and that are BRD1 target genes (*n* = 18 nMT genes). These 18 genes were significantly enriched for binding sites of the Peroxisome proliferator-activated receptor proteins PPARγ (*p* = 0.02) and PPARδ (*p* = 0.03) as well as two subunits of the nuclear transcription factor Y (NF-Y) (NF-Yα; *p* = 0.001 and NF-Yβ; *p* = 0.0005) (Supplementary Table S4). The NF-Y complex binds near DNA hormone response elements (HREs). Here it serves as a pioneer factor by promoting chromatin accessibility. PPAR, on the other hand, is a nuclear receptor that, upon activation by steroid hormones and various other lipid-soluble ligands, regulate the expression of HRE-containing genes [48]. Both NF-Y and PPAR have well-described roles in regulating mitochondrial function [49–51]. Interestingly, BRD1 contains four LXXLL signature motifs found in the majority of nuclear receptor co-activators [52, 53] and additionally, a CoRNR box often found in nuclear receptor co-repressors [54] (Fig. 3A).

To investigate whether BRD1 has the potential to modulate the genomic actions of PPARs and the transcription of HRE-containing genes in general, we used the CRISPR/Cas9 system to generate four lines of heterozygous *BRD1* knock out (BRD1^CRISPRex6/+^) HEK293T cells (clone 1–4) (Supplementary Fig. S3), and tested their ability to drive transcription of HRE-containing genes using a reporter array. We found that transcription mediated by Hepatocyte nuclear factor (HNF4, *p* = 0.046) and Androgen receptor (AR, *p* = 0.037) was significantly increased in BRD1^CRISPRex6/+^ cells, whereas transcription mediated by Glucocorticoid receptor (GR, *p* = 0.042) and Vitamin D receptor (VDR, *p* = 0.030) was significantly decreased (Fig. 3B). In addition, and in agreement with our in silico analysis, we saw a relatively large and close to significant increase in PPAR-mediated transcription (*p* = 0.085) (Fig. 3B), suggesting that BRD1 might be a co-repressor of PPAR-mediated transcription. This finding was replicated in an independent experiment (Supplementary Fig. S4). However, a particularly high variability was seen in measures of PPAR-mediated transcription, both in the BRD1^CRISPRex6/+^ and the WT group.

### Overexpression of BRD1-L affects mtDNA content and mitochondrial mass

**Fig. 4:**
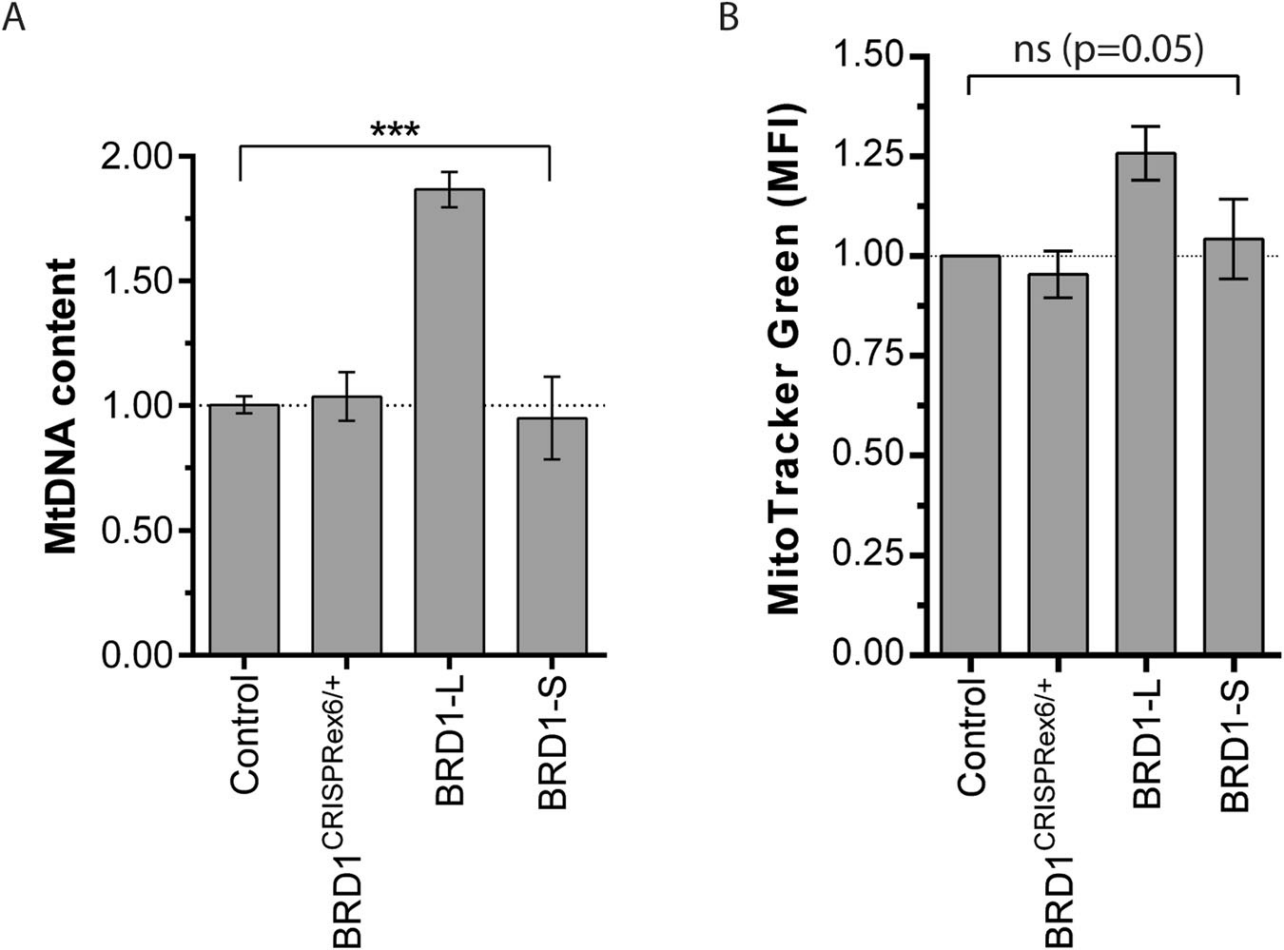
Investigation of the effect of altered BRD1 expression on mitochondrial amount. **A** Mitochondrial DNA content relative to the mean of two nuclear reference genes (*GAPDH* and *SLC34A2*) (*n* = 3/group). b Mitochondrial mass (*n* = 3/group). Control: naïve HEK293T cells, BRD1^CRISPRex6/+^: Clone #3 of BRD1^CRISPRex6/+^ HEK293T cells, BRD1-L: HEK293T cells stably overexpressing the BRD1-L isoform, BRD1-S: HEK293T cells stably overexpressing the BRD1-S isoform. Data presented as mean ± SEM. *P*-value as determined using ANOVA (brackets). *p* < 0.001 (***); not significant (ns).

Given the role of BRD1 as a potential regulator of nMT transcripts, we hypothesized that modulation of BRD1 levels, irrespective of whether they include underexpression of *BRD1* or overexpression of either of the two BRD1 isoforms (BRD1-L or BRD1-S), would influence mitochondrial biology. Hence, we investigated mitochondrial amounts and function in naïve HEK293T (control), BRD1-L, BRD1-S and BRD1^CRISPRex6/+^ (clone 3 with the lowest *BRD1* expression (Supplementary Fig. S3)) cell lines that all displayed comparable viability and growth rates (Supplementary Figs. S5–S6). Indicative of mitochondrial functional changes, we found a significant difference in mtDNA content between cell lines (ANOVA, *p* < 0.001) (Fig. 4A). BRD1-L appeared to have increased mtDNA content compared to control (1.87 ± 0.07), while BRD1^CRISPRex6/+^ and BRD1-S cells did not show differences in mtDNA content (Fig. 4A). Further indicating that BRD1-L cells have more and/or larger mitochondria, we observed a strong tendency towards a higher relative mitochondrial mass of 1.21 ± 0.07 in BRD1-L cells compared to the control (ANOVA, *p* = 0.05) (Fig. 4B).

### Overexpression of BRD1-S and BRD1–L affect reducing power of cells but not their calcium buffering capacity

**Fig. 5:**
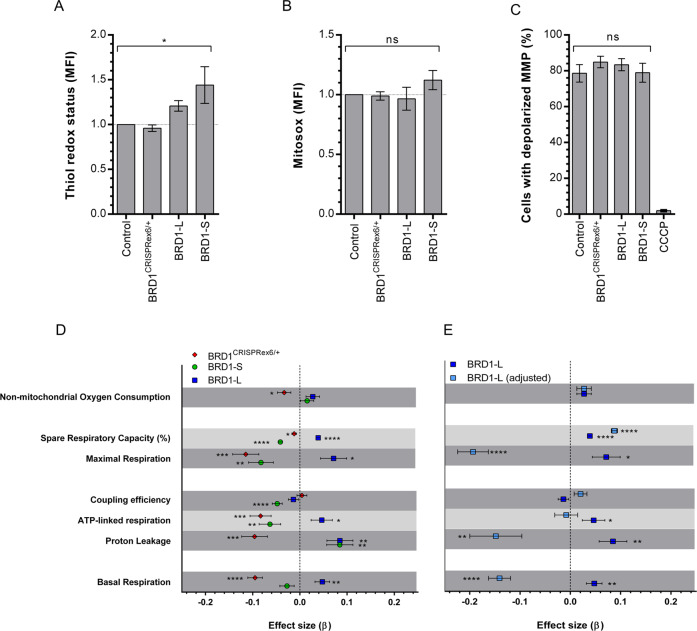
Investigation of the effect of altered *BRD1* expression on mitochondrial function. **A** Thiol redox status (*n* = 3/group). **B** Mitochondrial superoxide levels (*n* = 3/group). **C** Mitochondrial membrane potential (MMP) (*n* = 3/group). Cells treated with CCCP were used as negative controls. **A**–**C** Control: naïve HEK293T cells, BRD1^CRISPRex6/+^: Clone #3 of BRD1^CRISPRex6/+^ HEK293T cells, BRD1-L: HEK293T cells stably overexpressing the BRD1-L isoform, BRD1-S: HEK293T cells stably overexpressing the BRD1-S isoform. Data presented as mean ± SEM. *P*-value as determined by ANOVA (brackets). Mean fluorescence intensity (MFI). **D** Fundamental parameters of mitochondrial respiration in BRD1^CRISPRex6/+^, BRD1-L, and BRD1-S cell lines based on two independent measurements (*n* > 8/measurement). **E** Fundamental parameters of mitochondrial respiration in BRD1-L cell line, with and without adjustment for mtDNA content. **D**, **E** BRD1^CRISPRex6/+^: Clone #3 of BRD1^CRISPRex6/+^ HEK293T cells, BRD1-L: HEK293T cells stably overexpressing the BRD1-L isoform, BRD1-S: HEK293T cells stably overexpressing the BRD1-S isoform. *P*-value as determined using a linear mixed-effect model. Values were calculated on log10-transformed data and presented as β ± SE relative to the control (naïve HEK293T cells) (set 0). *p < 0.05; **p < 0.01; ***p < 0.001; ****p < 0.0001; not significant (ns).

Next, we evaluated the effect of decreased *BRD1* levels or increased levels of either of the two BRD1 isoforms on mitochondria-related processes in the same cell lines. First, we estimated mitochondrial oxidative stress levels by measuring levels of mitochondrial superoxide and second, we estimated cellular oxidative stress levels by measuring thiol redox status (TRS). Whereas we did not observe any statistically significant differences in mitochondrial superoxide levels between cell lines (ANOVA, *p* = 0.38) (Fig. 5B), we did observe significantly increased amounts of reduced thiols in both of the BRD1 overexpressing cell lines (BRD1-S 1.44 ± 0.20 and BRD1-L 1.21 ± 0.06, ANOVA *p* < 0.05) (Fig. 5A). In contrast, the amount of reduced thiols remained unchanged in BRD1^CRISPRex6/+^ cells (0.96 ± 0.04) (Fig. 5A). Increased TRS in the BRD1 overexpression cell lines could indicate an increased activated redox response system to maintain reactive oxygen species (ROS) homeostasis, resulting in unchanged mitochondrial superoxide levels as observed. We did not observe any difference in mitochondrial membrane potential (ANOVA, *p* = 0.66) (Fig. 5C), indicating that the mitochondria are intact in all four cell lines.

Rapid calcium influx through plasma membrane receptors and voltage-dependent ion channels in neurons plays a key role in neurosignaling [55]. Because high doses of calcium for extended periods are toxic to the cell, calcium needs to be buffered, among others by mitochondria. However, we did not observe any gross differences in mitochondrial calcium buffering capacity between cell lines (Supplementary Fig. S7).

### BRD1 levels affect mitochondrial respiration in an isoform-dependent manner

As we found enrichment for genes involved in the mitochondrial electron transport chain among the subset of nMT genes negatively correlated with *BRD1* expression in the brain, we measured the effect of BRD1 levels on oxygen consumption rates (OCR) reflecting mitochondrial electron transport and oxidative phosphorylation function, using the XFe96 extracellular flux analyzer. Based on these data, we calculated the effect size (β) of hampered and increased BRD1 levels relative to the control cell line (naïve HEK293T cells) (β = 0) on the following parameters: Basal respiration; ATP-linked respiration; Coupling efficiency; Maximal respiration; Non-mitochondrial oxygen consumption; Proton leakage; and Spare respiratory capacity (%) (Fig. 5D, Supplementary Fig. S1, Supplementary Table S3). Basal respiration was reduced in BRD1^CRISPRex6/+^ cells, increased in BRD1-L cells, and stable in BRD1-S cells. BRD1^CRISPRex6/+^ cells also had reduced non-mitochondrial respiration compared to control cells, while this remained constant in BRD1-L and BRD1-S cells. Interestingly, the coupling efficiency of BRD1^CRISPRex6/+^ and BRD1-L cells was unchanged compared to control, while the coupling efficiency of BRD1-S was reduced. BRD1-S cells seem to have a less efficient respiratory chain with reduced ATP-linked respiration, maximal respiration, and spare respiratory capacity, similar to BRD1CRISPRex6/+ cells. On the other hand, BRD1-L cells showed a more efficient mitochondrial electron transport chain with increased ATP-linked respiration, maximal respiration, and spare respiratory capacity.

However, since the increased basal respiration and increased spare capacity in BRD1-L without a change in coupling efficiency could be the result of the presence of more/larger mitochondria in BRD1-L cells (Fig. 4A, B), we adjusted the OCR values (except OCR values after addition of rotenone/antimycin A, as this is a non-mitochondrial parameter) by the relative mtDNA content (as a proxy for mitochondria content) and recalculated the parameters (Fig. 5E). After this adjustment, basal respiration (β = −0.14 ± 0.02, *p* < 0.0001), maximal respiration (β = −0.19 ± 0.03, *p* = 0.004), and proton leakage (β = −0.15 ± 0.05, *p* = 0.005) were reduced in BRD1-L cells compared to controls. On the other hand after adjustment, ATP-linked respiration was no longer significantly increased compared to controls (β = −0.01 ± 0.02, *p* = 0.7), while coupling remained unchanged. Taken together, these data suggest that BRD1-L cells might compensate for reduced basal respiration by promoting more/larger mitochondria or vice versa. Eukaryotic cells produce energy by oxidative phosphorylation and glycolysis, and most cells possess the ability to switch between these two pathways depending on changing environment. To evaluate whether cells experiencing dysfunctions in oxidative phosphorylation would produce energy by glycolysis instead, we measured changes in the extracellular acidification rates (ECAR) that result from the conversion of pyruvate generated by glycolysis to lactate. BRD1^CRISPRex6/+^ cells had reduced ECAR values compared to control, indicating more quiescent cells. BRD1-L cells had slightly increased ECAR levels, indicating that the cells are more energetic, whereas the ECAR levels of BRD1-S cells remained unchanged compared to the controls (Supplementary Fig. S8). These data suggest that no metabolic switch from mitochondrial respiration to glycolytic lactate takes place in conditions with reduced or increased BRD1 levels. On the contrary, it reflects an overall change in energy metabolism, where the BRD1^CRISPRex6/+^ cells become hypometabolic and the BRD1-L cells hypermetabolic, while the BRD1-S cells remain unchanged. A summary of the molecular, cellular, and mitochondrial characteristics of the BRD1^CRISPRex6/+^, BRD1-L, and BRD1-S cell lines is given in Table 1.

**Table 1:** Overview of molecular, cellular, and mitochondrial characteristics of the BRD1^CRISPRex6/+^, BRD1-L, and BRD1-S cell lines.

		BRD1^CRISPRex6/+^	BRD1-L (adjusted)	BRD1-S
*BRD1* expression	Relative levels of *BRD1* to *HPRT* and *PGK1* cDNA by qPCR	Decreased	Increased	Increased
Cell viability	Ratio viable/total cell count by IC with acridine orange and DAPI staining	NC	NC	NC
Growth rate	Cell count after 16 and 40 h by IC with acridine orange and DAPI staining	NC	NC	NC
MtDNA content	Relative levels of mtDNA to *GAPDH* and *SLC34A2* DNA by qPCR	NC	Increased	NC
Mitochondrial mass	MFI by IC with MitoTracker Green staining	NC	Increased*	NC
Amounts of reduced thiols	MFI by IC with VitaBright-48, propidium iodide, and acridine orange staining	NC	Increased	Increased
Superoxide levels	MFI by IC with MitoSOX and Hoechst staining	NC	NC	NC
Mitochondrial membrane potential	MFI by IC with JC-1 and DAPI staining	NC	NC	NC
Calcium buffering capacity	RFU after Ca^2+^ addition by microplate fluorescent readings with Calcium Green-5 N staining	NC	NC	NC
Basal respiration	XF Cell Mito Stress Test: T_3_–T_9_ with FCCP + oligomycin	Reduced	Increased (reduced)	NC
Proton leakage	XF Cell Mito Stress Test: T_4_–T_9_ with oligomycin	Reduced	Increased (reduced)	Increased
ATP-linked respiration	XF Cell Mito Stress Test: T_3_–T_4_ with oligomycin	Reduced	Increased (NC)	Reduced
Coupling efficiency	XF Cell Mito Stress Test: ATP-linked respiration/basal respiration with oligomycin	NC	NC (NC)	Reduced
Maximal respiration	XF Cell Mito Stress Test: T_4_–T_9_ with FCCP	Reduced	Increased (reduced)	Reduced
Spare respiratory capacity	XF Cell Mito Stress Test: maximal respiration/basal respiration with FCCP	Reduced	Increased (increased)	Reduced
Non-mitochondrial oxygen consumption	XF Cell Mito Stress Test: T_9_ with FCCP + oligomycin	Reduced	NC (NC)	NC
Basal energy metabolism	OCR/ECAR ratio	Reduced	Increased	NC

*DAPI* 4′,6′-diamidino-2-phenylindole, *ECAR* extracellular acidification rate, *FCCP* trifluoromethoxy carbonylcyanide phenylhydrazone, *IC* image cytometry, *MFI* median fluorescence intensity, *NC* no chance, *OCR* oxygen consumption rate, *qPCR* quantitative real-time PCR, *RFU* relative fluorescence units.

*p = 0.05 (not significant).

## Discussion

Mitochondria are subcellular organelles that harbor essential functions such as energy metabolism, redox signaling, and Ca^2+^ buffering. Long lists of disorders associated with mitochondrial dysfunction underline the importance of proper mitochondrial functioning in health [56–58]. Typically, tissues with high-energy demands are affected by mitochondrial dysfunction, like the brain which requires up to 20% of the total-body energy while only accounting for ~2% of the body mass [59].

Several lines of evidence suggest that mitochondrial dysfunction is an important component in the neurobiology of neuropsychiatric and neurodevelopmental disorders [60–68]. These include findings from genetic [69–71], brain-imaging [72, 73], biomarker, and post-mortem brain studies [74–76]. Particularly, dysregulation of nMT genes has been reported in bipolar disorder [77, 78], SZ [79–83], and ASD [84]. This has led to the suggestion that neurons of patients with neuropsychiatric or neurodevelopmental disorders may be less tolerant to challenges normally dealt with by mitochondria, such as increased oxidative stress or high energy demand, hereby likely affecting neuronal characteristics that mitochondria play a key role in, such as neurogenesis, neuromorphology, and neuroactivity [69, 83, 85]. A recent study investigating mitochondrial function in induced pluripotent stem cell-derived neurons from patients even suggested enhancement of mitochondrial biogenesis as a treatment for SZ [86].

Here, we followed up on transcriptomic and proteomic evidence implying mitochondrial dysfunction in the brain of mice with reduced expression of the psychiatric risk gene *Brd1*. Using publicly available datasets, we demonstrate that *BRD1* expression levels are negatively correlated to nMT gene expression levels in the developing human brain, and that deviation from standard physiological BRD1 levels, regardless of direction and BRD1 isoform, leads to a significant shift in the expression profile towards a negative fold change of nMT genes in vitro. Interestingly, and despite this overall similarity in the shift of the expression profiles of nMT genes, overexpression of either of the individual BRD1 isoforms or downregulation of *BRD1* overall affect the expression of distinct sets of nMT genes, and generally with opposing expression changes observed between BRD1 depleted and BRD1 overexpressing cells.

Providing some mechanistic insight into how BRD1 may regulate the transcription of nMT genes, we demonstrate that known members of BRD1 protein complexes, DNMT1 and KMT5B [23, 24], display similar nMT gene co-expression characteristics as BRD1. Both DNMT1 and KMT5B are involved in the repression of gene expression by epigenetic modifications [87]; however, additional experimental evidence besides these limited observational expression correlations are warranted to reveal how BRD1 specifically regulates nMT genes. We furthermore identify NF-Yα, NF-Yβ, PPARδ, and PPARγ as likely co-factors of BRD1 in the direct regulation of nMT genes. NF-Yα and NF-Yβ, two subunits of the CBF/NF-Y transcription factor, bind in close proximity to HRE binding sites, such as Progesterone receptor (PGR), Glucocorticoid receptor (GR) and Androgen receptor (AR), and regulate gene expression of gene clusters, including nMT genes [49]. Similarly, PPARγ and PPARδ have well-described roles in the regulation of mitochondrial function and PPAR-γ has even been investigated as a therapeutic target for mental disorders in this regard [50, 51]. Moreover, KMT5B and PPARγ complexes have been shown to govern metabolic processes [88]. We provide experimental evidence that BRD1 is a co-regulator of nuclear receptor-mediated gene transcription. Particularly, we show that hampered BRD1 expression is associated with increased PPARγ facilitated transcription, suggesting that BRD1 is a co-repressor of PPARs.

*BRD1* expression levels affect the expression of genes related to mitochondrial energy metabolism in human brain, more specifically the electron transport chain including respiratory chain complex I, and reduced BRD1 levels result in reduced mitochondrial respiration in the BRD1^CRISPRex6/+^ cell line. The latter is more likely a consequence of reduced respiratory chain activity or respiratory chain complex I proteins, rather than dysfunctional respiratory chain complexes or a reduction in number of mitochondria since neither coupling efficiency, mtDNA content, nor mitochondrial mass are altered in the BRD1^CRISPRex6/+^ cell line. Interestingly, overexpression of the BRD1-L isoform enhances bioenergetics capacity by increasing both respiration and glycolytic lactate production. As this increase in respiration occurs without altering the coupling efficiency, it might likely be the result of more and/or larger mitochondria per cell. Yet, this is not the case when overexpressing the BRD1-S isoform, where the basal bioenergetics capacity is unchanged, despite a reduced coupling efficiency and reduced ATP-linked respiration and spare capacity. The increased proton leakage observed when BRD1-L and BRD1-S are overexpressed, and the reduced coupling efficiency observed in BRD1-S cell lines could lead to increased ROS production by the respiratory chain, the major source of ROS in the cell [89]. We indeed observed an activation of the ROS scavenging systems as measured by increased levels of reduced thiols in the BRD1-L and BRD1-S cell lines, which could restore the mitochondrial redox homeostasis in these cells. Thus, deviations from standard physiological BRD1 levels seem in general to affect mitochondrial function, yet in distinct ways depending on whether *BRD1* is underexpressed or the one or the other BRD1 isoform is upregulated. This might be based in different gene set modulations taking place in the different cell lines. The differences between isoforms are important for any potential treatment that would target BRD1, as the mechanisms underlying differential regulation of BRD1 isoform expression are currently unknown. The synergy between overexpression of either of the BRD1 isoforms and downstream effects in the cell remains to be studied.

A general and important limitation of our study is also that all experimental findings are derived from immortalized HEK293T cell lines and that only one line per condition was investigated. Although our studies specifically took its starting point in cell lines with well-characterized modulations of a psychiatric disorders risk gene, further validation of an association between BRD1 and mitochondrial dysfunction as well as its link to psychiatric disorders is warranted. This could be provided by future studies in genetically modified neuronal cell lines, in brain tissue from mouse models, or in patient-derived cells with disrupted BRD1 function.

In this line, dysfunctional mitochondrial bioenergetics could explain some of the previously observed phenotypes in the brains of *Brd1*^*+/−*^ mice. For example, parvalbumin-positive GABAergic interneurons and medium spiny neurons are neuron types that are well known to display particularly high energy demand and sensitivity to energy imbalances and these are the neurons selectively lost in the brains of *Brd1*^*+/−*^ mice as early as in newborn pups (P0) [33]. Neurons from *Brd1*^*+/−*^ mice that do survive into adulthood have reduced dendritic arborization and spine density and aberrant spine morphology, phenotypes that have previously been linked to mitochondrial dysfunction and that could lead to cortical under-connectivity and cognitive impairments [34, 90–92]. The subsequent disturbances in the excitatory/inhibitory balance and improper signaling, networking, and brain functioning provide a plausible explanation of the role of BRD1 in psychopathology.

Of particular importance in the understanding of the molecular, cellular, and clinical manifestations of BRD1 deficiency in humans, it has recently been shown that the *BRD1* gene is the only fully contained protein-coding gene in a genomic region (Chr22:49238268–50248907/hg19), that seems critical in the formation of a 22q13.3 large-deletion and Phelan-McDermid syndrome specific genome-wide DNA methylation epi-signature [18]. Candidate effectors of this epi-signature could indeed be the BRD1 interactions partners and methyltransferases, DNMT1 and KMT5B as for the reasons described above. Of special note, and well in line with our findings that cells with reduced amount of BRD1 (the BRD1^CRISPRex6/+^ cell line) are mitochondrially compromised and hypometobolic, lymphoblastoid cell lines from individuals with this Phelan-McDermid syndrome DNA methylation epi-signature (when compared to cells from epi-signature negative individuals with small 22q13.3 deletions sparing *BRD1*) show significantly different and specific metabolic profiles characterized by reduced energy production in the presence of high-efficiency energy sources, decreased ability of metabolic adjustment to environmental changes, as well as abnormal responses to hormones and cytokines regulating energy storage, proliferation, growth, and inflammation [18]. Thus, *BRD1* might be responsible for a phenotypically distinct clinical subtype of Phelan McDermid syndrome. Specific evidence of respiratory chain dysfunction in Phelan-McDermid syndrome, especially involving the complex I and IV, has previously been demonstrated in patient-derived buccal swaps [93]. Of note, in that study, 13 out of 22 samples carrying *BRD1* deletions also had respiratory chain complex abnormalities. We further observe in the study by Schenkel et al. [18] that not only the *BRD1* gene itself is differentially methylated (10-14% increased methylation as determined by three probes close to the transcription start site) in the absence of one copy of *BRD1* but also the *PPARG* gene at chromosome 3 encoding PPARγ shows 13% decreased methylation (as determined by one probe in the *PPARG* 5′ UTR). Unfortunately, the study of the Phelan-McDermid syndrome cells did not include genome-wide gene expression measurements, neither did our own study of the BRD1^CRISPRex6/+^ cell line. However, indeed the BRD1-KD cells [23] show a 1.8 fold increased expression of *PPARG* and the BRD1^CRISPRex6/+^ cell line show a more than the expected 50% decrease in *BRD1* expression. We thus speculate, based in our joint findings, that heterozygosity for a *BRD1* gene deletion/inactivation not only leads to further epigenetic downregulation of the remaining copy of *BRD1* but also to metabolic/mitochondrial dysfunction due to, at least partly, downstream epigenetic effects of BRD1 deficiency on the expression of *PPARG*.

In conclusion, we show that BRD1 regulates the expression of genes important for mitochondrial function, likely through its interaction with selected transcription factors and epigenetic modifiers, or through indirect modulation of mitochondrial biology. We furthher show that proper function of mitochondria seems to be dependent on a balanced expression of *BRD1* and changes in BRD1 levels lead to aberrant mitochondrial bioenergetics in an isoform-specific manner. Mitochondrial dysfunction provides a possible mechanistic explanation for the phenotypes observed in *Brd1*^+/−^ mice and a conceivable link between genetic variation in *BRD1* and psychopathologies in humans. Since mitochondrial modulators are emerging as an effective alternative treatment paradigm in psychiatric disorders [94], future studies in *BRD1*-based cell and mouse models should address both short- and long-term effects of such drugs as well as their underlying mechanisms of action.

## Supplementary information

Supplementary information

Table S1
