# Supplementary material for: The psychiatric risk gene BRD1 modulates mitochondrial bioenergetics by transcriptional regulation
Source: Transl Psychiatry. 2022 Aug 8;12:319. doi: 10.1038/s41398-022-02053-2 (PMC9359996; doi:10.1038/s41398-022-02053-2)
Supplement: Supplementary file 1 — Supplementary information [file 41398_2022_2053_MOESM1_ESM.docx]

**Supplementary information**

**The Psychiatric Risk Gene BRD1 Modulates Mitochondrial Bioenergetics by Transcriptional Regulation**

Veerle Paternoster^1,2,3,4^, Cagla Cömert^5^, Louise Sand Kirk^1,2,3,4^, Sanne Hage la Cour^1,2,3,4^, Tue Fryland^1,2,3,4^, Paula Fernandez-Guerra^5^, Magnus Stougaard^6,7^, Jens Randel Nyengaard^8^, Per Qvist^1,2,3,4🖂^, Peter Bross^5^, Anders Dupont Børglum^1,2,3,4^, and Jane Hvarregaard Christensen^1,2,3,4🖂^

^1^The Lundbeck Foundation Initiative for Integrative Psychiatric Research, iPSYCH, Aarhus, Denmark

^2^Centre for Integrative Sequencing, iSEQ, Aarhus University, Aarhus, Denmark

^3^Department of Biomedicine, Aarhus University, Aarhus, Denmark

^4^Center for Genomics and Personalized Medicine, CGPM, Aarhus University, Aarhus, Denmark

^5^Research Unit for Molecular Medicine, Department of Clinical Medicine, Aarhus University and Aarhus University Hospital, Aarhus, Denmark

^6^Department of Pathology, Aarhus University Hospital, Aarhus, Denmark

^7^Department of Clinical Medicine, Aarhus University, Aarhus, Denmark

^8^Core Center for Molecular Morphology, Section for Stereology and Microscopy, Department of Clinical Medicine, Aarhus University, Aarhus, Denmark

**^🖂^Corresponding authors:**

Per Qvist, PhD and Jane H. Christensen, PhD

Department of Biomedicine, Aarhus University

Høegh-Guldbergs Gade 10, Building 1116

DK-8000 Aarhus C, Denmark

Phone: +45 50192009 / +45 93522003

Email: per.q@biomed.au.dk and jhc@biomed.au.dk

**Table of Contents**

[Supplementary Methods 3](#_Toc103590976)

[Generation of BRD1 knockdown cell lines by CRISPR/Cas9 3](#_Toc103590977)

[a. Transfection of HEK293T cells 3](#_Toc103590978)

[b. DNA isolation and Sanger sequencing 3](#_Toc103590979)

[c. Quantitative Real-time PCR 3](#_Toc103590980)

[d. Western blotting 3](#_Toc103590981)

[Supplementary Figures 5](#_Toc103590982)

[Figure S1: Schematic overview of the experimental output of bioenergetics measurements 5](#_Toc103590983)

[Figure S2: Spatiotemporal analysis 6](#_Toc103590985)

[Figure S3: BRD1 expression levels in genetically modified HEK293T cells 7](#_Toc103590986)

[Figure S4: Dual luciferase assay of transcription from hormone response elements (HREs) 8](#_Toc103590987)

[Figure S5: Cell viability of cell lines 9](#_Toc103590988)

[Figure S6: Growth rates of cell lines 10](#_Toc103590989)

[Figure S7: Calcium Green-5N fluorescence in isolated mitochondria from cell lines 11](#_Toc103590990)

[Figure S8: Basal oxygen consumption rate to extracellular acidification rate ratio in cell lines 12](#_Toc103590991)

[Supplementary Tables 13](#_Toc103590992)

[Table S1: Gene lists (online file) 13](#_Toc103590993)

[Table S2: Primer sequences used for quantitative Real-time PCR 14](#_Toc103590994)

[Table S3: Calculation of bioenergetics parameters 15](#_Toc103590995)

[Table S4: Enrichment of transcription factor binding 16](#_Toc103590996)

[References 17](#_Toc103590997)

# Supplementary Methods

## Generation of BRD1 knockdown cell lines by CRISPR/Cas9

### a. Transfection of HEK293T cells

Three sets of sgRNA were designed using the CRISPR Design website: crispr.mit.edu to target exon 6 of *BRD1*. Assessment of resulting changes to protein sequence with the ExPASy Translate tool (<http://web.expasy.org/translate/>) was applied to confirm allelic disruption. sgRNAs were cloned into the pSpCas9(BB)-2A-Puro (PX459) V2.0 plasmid (Addgene plasmid 62988) [1] following the general cloning protocol from Zhang lab (<http://www.genome-engineering.org/>). HEK293T cells (ATCC, Manassas, VA) were cultured in Dulbecco’s modified Eagle’s medium (DMEM) (Lonza, Verviers, Belgium) supplemented with 10% fetal calf serum, 2 mM L-glutamine and 50 mg/L penicillin/streptomycin (Sigma-Aldrich).

Cells were transfected with one of the three different sgRNA plasmids or a GFP transfection control (pEGFP-C1) using X-tremeGENE 9 DNA transfection reagent (Roche Applied Science, Penzberg, Germany) according to the manufacturer’s protocol. On the following day, puromycin (1 μg/ml) (InvivoGen, San Diego, CA, USA) was added for selection for 12 days. Cells were harvested after incubation with trypsin, washed and pelleted by centrifugation for further use.

### b. DNA isolation and Sanger sequencing

Cell pellets were dissolved in 300 μl lysis buffer (Roche) and 30 μl proteinase K (Finnzymes, Espoo Finland). The reaction was left at 56°C for 1 hour and processed using the Maxwell® 16 blood DNA purification kit (Promega) according to the manufacturer’s protocol. The efficiency of the individual sgRNA’s and INDELs were investigated by PCR using the Q5® Hot Start High-Fidelity DNA Polymerase (NEB) according to the manufacturer’s protocol. 10 pmol of each primer and 50 ng genomic DNA were used and the PCR cycling conditions were as follows: Initial denaturation at 98°C for 30 sec. followed by 35 cycles with denaturation at 98°C for 10 sec., annealing at 59°C for 30 sec., extension at 72°C for 30 sec. and a final extension at 72°C for 2 min. The PCR products were separated by electrophoresis on a 4% Agarose gel and purified with QIAquick® Gel Extraction kit (QIAGEN) according to the manufacturer’s protocol. The purified PCR fragments were sequenced by GATC Biotech AG (Germany) and analyzed by Tracking of Indels by DEcomposition (TIDE) software [2].

### c. Quantitative Real-time PCR

Downregulation of *BRD1* in selected clones was confirmed by quantitative Real-time PCR as described in the main text.

### d. Western blotting

Cell pellets were dissolved in Paul Fraser lysis buffer supplemented with 100 mg/mL Sodium Butyrate, 100 mM Sodium Orthovanadate and one cOmplete Tablet, Mini, EDTA-free, EASYpack (Roche) and subjected to repeated freeze/thaw cycles followed by centrifugation at 15,000 rpm for 5 min. Supernatants were collected and protein concentration measured by NanoDrop 1000 version 3.7.1 (Thermo Fisher Scientific, Inc., Waltham, MA, USA). Equal protein volumes were mixed with loading buffer and size-separated on a Criterion TGX Precast Gel (Bio-Rad Laboratories) before transfer on an Amersham Hybrid-P membrane (GE Healthcare, Waltham, MA, USA). The membrane was blocked in 10% milk in PBS and incubated with the following primary antibodies and appropriate secondary antibodies: Chicken α-BRD1 (1:1,000, Aves Labs, Inc., Tigard, US) and Rabbit α-Tubulin (1:10,000, Abcam, Cambridge, UK)), goat α-Chicken (1:10,000, Aves Labs) and goat α-mouse (1:1,000, Dako, Glostrup, Denmark). The membrane was developed using Supersignal Western Dura Extended Duration Substrate (Thermo Fisher Scientific) and captured by a Fujifilm LAS-4000 mini Imager (Fujifilm, Minato (Tokyo), Japan) using Image Quant^TM^, LAS 4000 mini v 1.2, (GE Healthcare Bio-Sciences Corp.). Densitometric analysis was done with ImageQuantTL v7.0 (GE Healthcare Bio-Sciences Corp.).

# Supplementary Figures


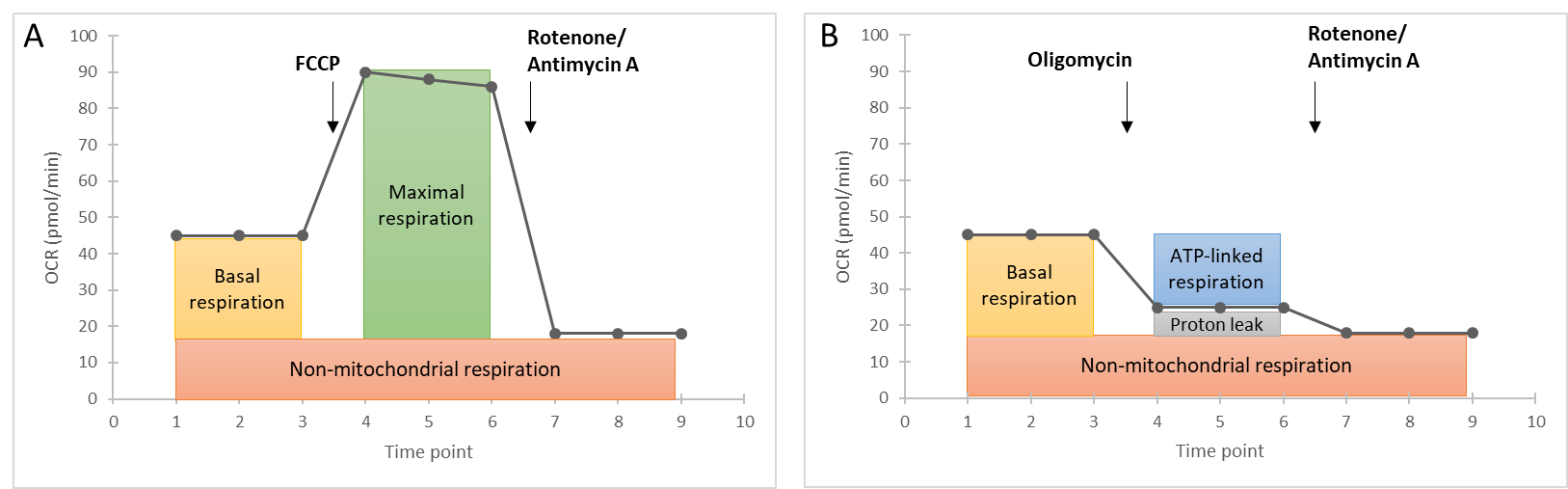


## Figure S1: Schematic overview of the experimental output of bioenergetics measurements

A) Schematic overview of oxygen consumption rates (OCR) profile after addition of the mitochondrial oxidative phosphorylation uncoupler, FCCP and mitochondrial respiration inhibitors, Rotenone/Antimycin A. B) Schematic overview of OCR profile after addition of the ATP synthase inhibitor, Oligomycin, and mitochondrial respiration inhibitors, Rotenone/Antimycin A.

### **
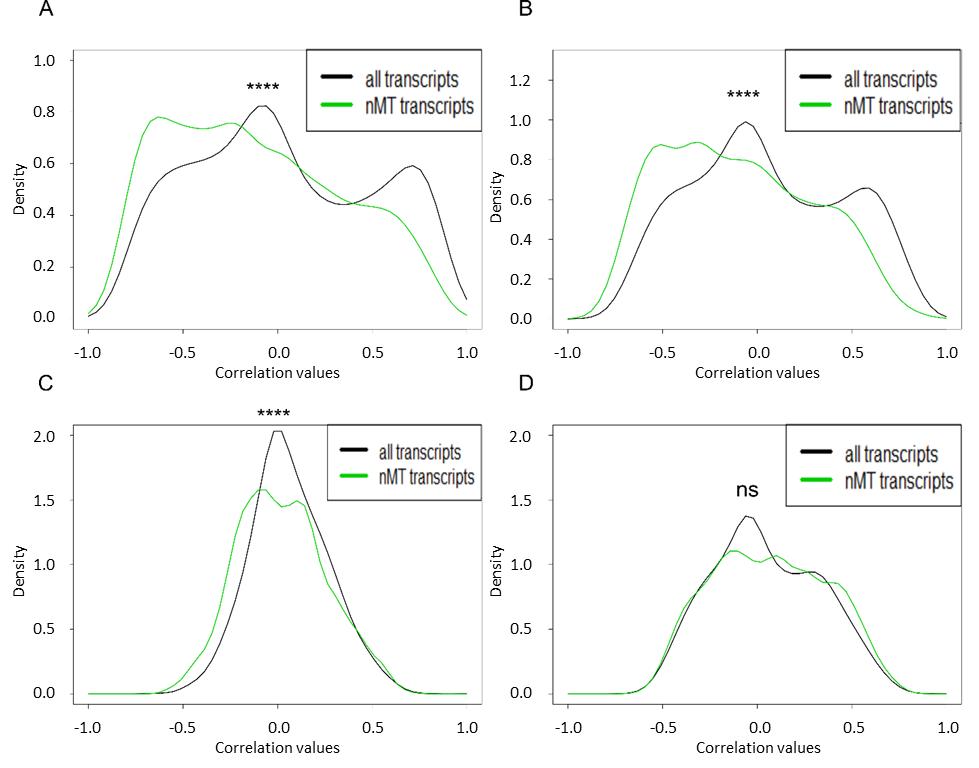
**

## Figure S2: Spatiotemporal analysis

Spatiotemporal analysis of the correlation between the expression of nuclear-encoded mitochondrial (nMT) transcripts and the expression of four protein interaction partners of BRD1. Correlation values for the nMT transcripts represent the level of correlation with: A) KMT5B (Δmode_nMT-all_ = -0.65, p = 1 x10^-20^) (****), B) DNMT1 (Δmode_nMT-all_ = -0.46, p = 6 x10^-29^) (****), C) KAT7 (Δmode_nMT-all_ = -0.13, p = 8 x10^-9^) (****) and D) KAT5 (Δmode_nMT-all_ = 0.05, p = 0.08) (ns) in the developing human brain.


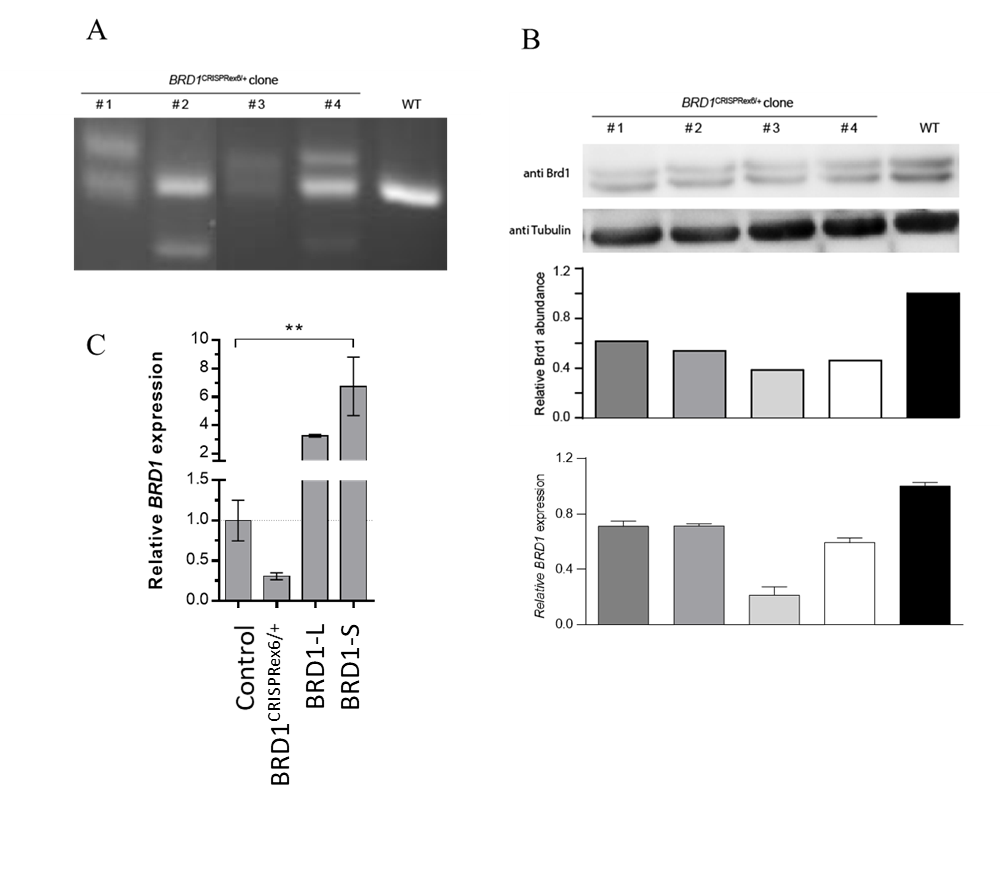


###

## Figure S3: BRD1 expression levels in genetically modified HEK293T cells

A) Gel electrophoresis of PCR products spanning the CRISPR/Cas9 target site reveals insertions and/or deletions in all selected clones. Irrelevant lanes have been removed from gel image. B) Reduced *BRD1* expression in selected BRD1^CRISPRex6/+^ HEK293T clones was confirmed by Western blotting analysis using antibodies directed against BRD1 and Tubulin. Quantification of the relative intensity (BRD1/Tubulin) show a consistent ~50% reduction in BRD1 abundancy in BRD1^CRISPRex6/+^ HEK293T cells. Quantification of the relative levels of *BRD1* mRNA to *HPRT* and *PGK1* mRNA by qPCR similarly confirms reduction in *BRD1* mRNA in all four BRD1^CRISPRex6/+^ HEK293T clones. C) Relative *BRD1* expression levels in modified HEK293T cell lines as measured by qPCR. Control: naïve HEK293T cells, BRD1^CRISPRex6/+^: Clone #3 of BRD1^CRISPRex6/+^ HEK293T cells, BRD1-L: HEK293T cells stably overexpressing the BRD1-L isoform, BRD1-S: HEK293T cells stably overexpressing the BRD1-S isoform. Data presented as mean ± SEM. P-value as determined by ANOVA (bracket). p < 0.01 (**).


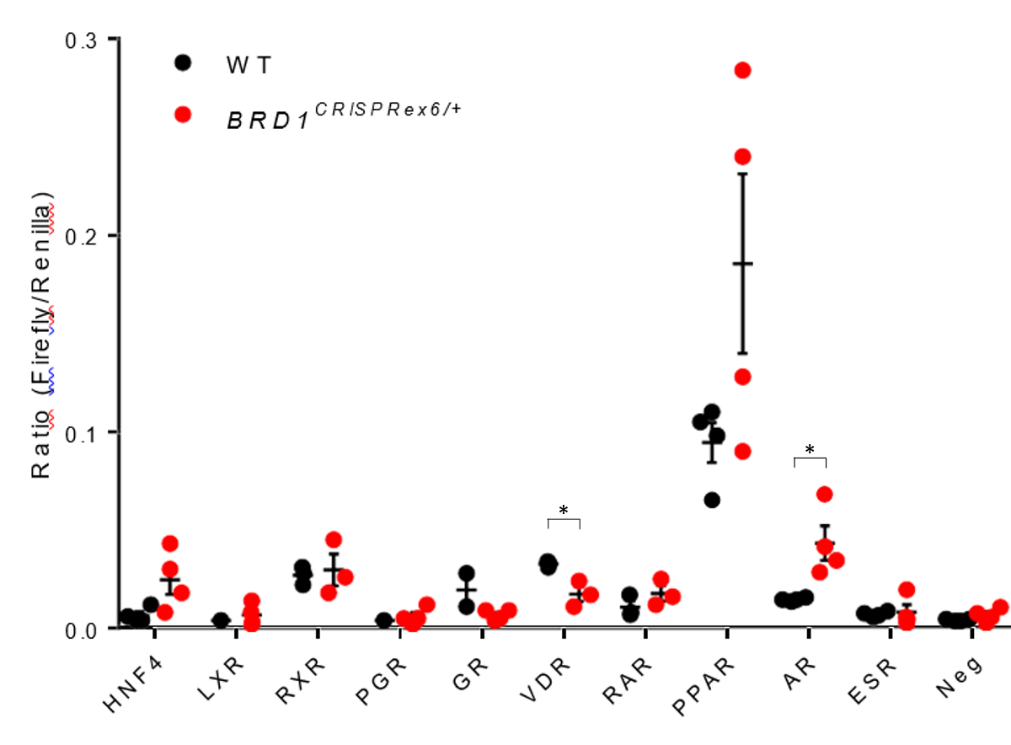


## Figure S4: Dual luciferase assay of transcription from hormone response elements (HREs)

Replication of the measures of transcription from promoters containing HREs were replicated on the same BRD1^CRISPRex6/+^ and WT colonies by a dual luciferase based array. P-values as determined by Student’s t-tests (brackets). p < 0.05 (*).


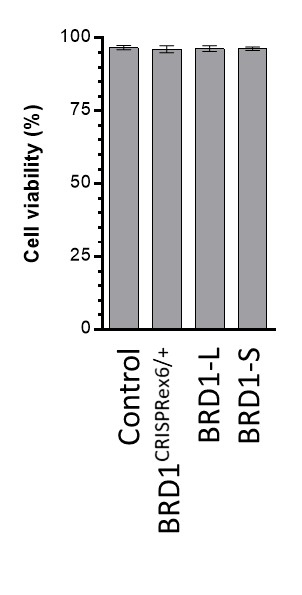


## Figure S5: Cell viability of cell lines

Cells were stained with Solution 13 (Chemometec), which contains two nuclear specific dyes: Acridine Orange to stain the entire population of cells, and 4′,6′-Diamidino-2-phenyl-indole (DAPI) to stain the non-viable cells. Cell viability was estimated as the ratio viable/total cell count (n=3/group). Control: naïve HEK293T cells, BRD1^CRISPRex6/+^: Clone #3 of BRD1^CRISPRex6/+^ HEK293T cells, BRD1-L: HEK293T cells stably overexpressing the BRD1-L isoform, BRD1-S: HEK293T cells stably overexpressing the BRD1-S isoform.


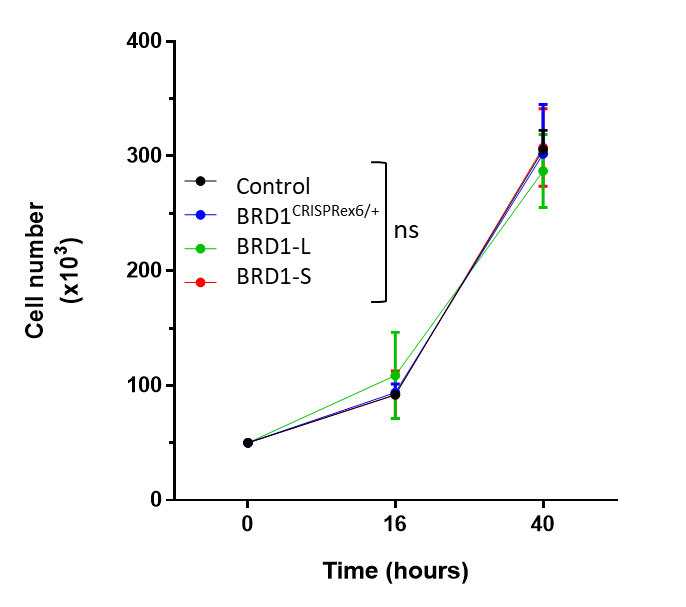


## Figure S6: Growth rates of cell lines

Cells (n=50,000) were seeded and counted after respectively, 16h and 40h (n=2 cultures/cell line). Control: naïve HEK293T cells, BRD1^CRISPRex6/+^: Clone #3 of BRD1^CRISPRex6/+^ HEK293T cells, BRD1-L: HEK293T cells stably overexpressing the BRD1-L isoform, BRD1-S: HEK293T cells stably overexpressing the BRD1-S isoform. Data presented as mean ± SEM. Statistical significance as determined by repeated measure ANOVA (bracket). Not significant (ns).


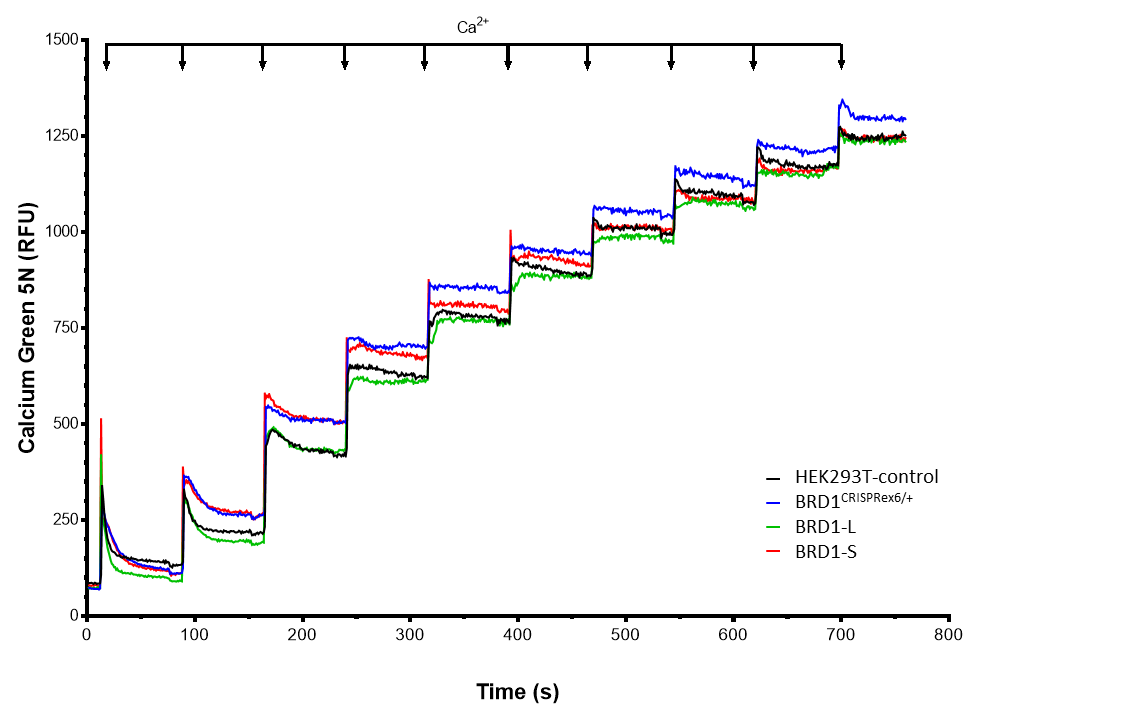


## Figure S7: Calcium Green-5N fluorescence in isolated mitochondria from cell lines

Calcium absorbance by isolated mitochondria was measured by repeatedly adding Ca^2+^ and measuring Calcium Green 5N fluorescence decay. The calcium absorbance pattern is similar for BRD1-control, BRD1-L, BRD1-S, and BRD1^CRISPRex6/+^ HEK293T cells indicating no difference between the cell lines. HEK293T-control: naïve HEK293T cells, BRD1^CRISPRex6/+^: Clone #3 of BRD1^CRISPRex6/+^ HEK293T cells, BRD1-L: HEK293T cells stably overexpressing the BRD1-L isoform, BRD1-S: HEK293T cells stably overexpressing the BRD1-S isoform.


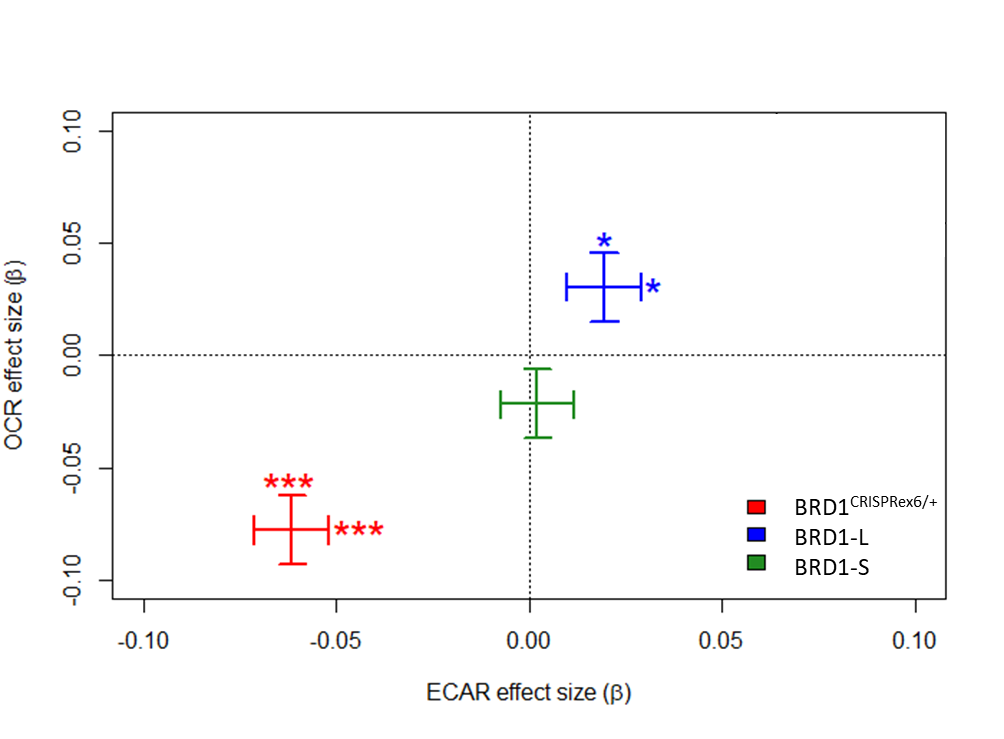


## Figure S8: Basal oxygen consumption rate to extracellular acidification rate ratio in cell lines

Basal oxygen consumption rate (OCR) to extracellular acidification rate (ECAR) ratio in BRD1^CRISPRex6/+^, BRD1-L, and BRD1-S cell lines relative to control (naïve HEK293T cells) (set 0) based on two independent measurements (n>8/measurement). BRD1^CRISPRex6/+^: Clone #3 of BRD1^CRISPRex6/+^ HEK293T cells, BRD1-L: HEK293T cells stably overexpressing the BRD1-L isoform, BRD1-S: HEK293T cells stably overexpressing the BRD1-S isoform. Data presented as effect size (β) ± SE relative to the control (set 0). P<0.05 (*), p<0.001 (***).

# Supplementary Tables

## Table S1: Gene lists (online file)

Gene lists downloaded from Ensembl, Brainspan, or Fryland et al. (2016) [3] are given. Annotations indicate identification of genes as nMT, BRD1-L targets, or BRD1-S targets. (1 = yes, 0 = no). Numbers on top of the columns indicate total number of genes partaking in the group.

## Table S2: Primer sequences used for quantitative Real-time PCR

| **Gene** | **Forward primer (5’-3’)** | **Reverse primer (5’-3’)** |
| --- | --- | --- |
| **cDNA** | | |
| *BRD1* | TGCATCGAGAATGGGAACTAC | ACCACCTTCAGAGGCTCCAG |
| *GAPDH* | TCTCCTCTGACTTCAACAGCGAC | CCCTGTTGCTGTAGCCAAATTC |
| *POLR2* | TGCCCGAGACAAGACTGGCT | CGCTTGCCCTCGACGTTCTG |
| *HPRT* | TGCTGAGGATTTGGAAAGGGTGT | CCTTGAGCACACAGAGGGCTA |
| *RPS* | GCCGGATTCACCGTTTGGCT | ATTTATGCGACCAGGGCAGAGG |
| *PGK1* | AAGCTGACGCTGGACAAGCTG | GATGCTTGGGACAGCAGCCTTA |
|  | | |
| **DNA** | | |
| *tRNA^Leu(UUR)^* (mitochondrial genome) | CACCCAAGAACAGGGTTTGT | TGGCCATGGGTATGTTGTTA |
| *GAPDH* | CCCGGTTTCTATAAATTGAGCCCGCA | AAAGAAGATGCGGCTGACTGTCG |
| *SLC34A2* | TCCCAAACGCAAATCCTTTAGA | CTCTCGGAACCACATTTCAAGATAA |
|  | | |
| **CRISPR knockout validation** | | |
| *BRD1* | GCCGAAGGTGATTTGAAAGA | TTTACGGAACTGGCG CTAAC |
|  | | |
| **sgRNA** | | |
| Brd1_CRISPR_#1 | CACCGGCTCAGCTTGTTTCGGAGAA | AAACTTCTCCGAAACAAGCTGAGCC |
| Brd1_CRISPR_#2 | CACCGGCCGCTGGACTTCATAGCGC | AAACGCGCTATGAAGTCCAGCGGCC |
| Brd1_CRISPR_#3 | CACCGTGGACTTCATAGCGCAGGTG | AAACCACCTGCGCTATGAAGTCCAC |

## Table S3: Calculation of bioenergetics parameters

| **Parameter** | **Calculation** | **Drug** |
| --- | --- | --- |
| Non-mitochondrial respiration | T_9_ | FCCP + Oligomycin |
| Basal respiration | T_3_ – T_9_ | FCCP + Oligomycin |
| Maximal respiration | T_4_ – T_9_ | FCCP |
| Spare capacity (%) | Maximal respiration / Basal respiration * 100% | FCCP |
| ATP-linked respiration | T_3_ – T_4_ | Oligomycin |
| Proton leakage | T_4_ – T_9_ | Oligomycin |
| Coupling efficiency | ATP-linked respiration / basal respiration * 100% | Oligomycin |

One time point (T) per interval of three repeated measurements was chosen according to the manufacturer’s guidelines. Time points (T) as indicated in **Suppl. Figure S1**.

## Table S4: Enrichment of transcription factor binding

| **Term** | **Study** | **Odds Ratio** | **Adjusted P-value** |
| --- | --- | --- | --- |
| NFYB | ENCODE | 8.79 | 0.0005 |
| NFYA | ENCODE | 7.92 | 0.001 |
| PPARG | CHEA | 10.47 | 0.02 |
| PPARD | CHEA | 13.97 | 0.03 |

Enrichment of transcription factor binding to nMT genes that are negatively correlated with the expression of *BRD1* and that are target genes of either BRD1-S or BRD1-L. P-value calculated by Fisher’s exact test adjusted for multiple testing by the Benjamini-Hochberg method.

# References

[1] F.A. Ran, P.D. Hsu, J. Wright, V. Agarwala, D.A. Scott, F. Zhang, Genome engineering using the CRISPR-Cas9 system, Nat. Protoc. 8 (2013) 2281–2308. doi:10.1038/nprot.2013.143.

[2] E.K. Brinkman, T. Chen, M. Amendola, B. van Steensel, Easy quantitative assessment of genome editing by sequence trace decomposition, Nucleic Acids Res. 42 (2014) e168–e168. doi:10.1093/nar/gku936.

[3] T. Fryland, J.H. Christensen, J. Pallesen, M. Mattheisen, J. Palmfeldt, M. Bak, J. Grove, D. Demontis, J. Blechingberg, H.S. Ooi, M. Nyegaard, M.E. Hauberg, N. Tommerup, N. Gregersen, O. Mors, T.J. Corydon, A.L. Nielsen, A.D. Børglum, Identification of the BRD1 interaction network and its impact on mental disorder risk., Genome Med. 8 (2016) 53. doi:10.1186/s13073-016-0308-x.
